# RNA modification of an RNA modifier prevents self-RNA sensing

**DOI:** 10.1371/journal.pbio.3001342

**Published:** 2021-07-30

**Authors:** Daltry L. Snider, Stacy M. Horner

**Affiliations:** 1 Department of Molecular Genetics & Microbiology, Duke University School of Medicine, Durham, North Carolina, United States of America; 2 Department of Medicine, Duke University School of Medicine, Durham, North Carolina, United States of America

## Abstract

This Primer explores a new study in PLOS Biology which finds that interferon-induced ADAR1 mRNA is m6A-modified to promote its translation, enabling ADAR1 to modify self-dsRNAs generated during the interferon response; this prevents recognition of these self RNAs and erroneous activation of the MDA5-mediated host antiviral response.

## Teaser

A new study in *PLOS Biology* finds that interferon (IFN)-induced adenosine deaminase acting on RNA 1 (*ADAR1*) mRNA is N6-methyladenosine (m^6^A) modified to promote its translation, enabling ADAR1 to modify self-double-stranded RNAs (dsRNAs) generated during the IFN response and preventing activation of the melanoma differentiation–associated protein 5 (MDA5)-mediated host antiviral response.

The discrimination between self- and foreign-derived nucleic acids, such as from viruses, is critical to allow for induction of type I interferon (IFN) while preventing autoinflammation. IFN is induced when cytosolic RNA pattern recognition receptors (PRRs) retinoic acid–inducible gene I (RIG-I) and melanoma differentiation–associated protein 5 (MDA5) sense specific patterns in RNA, which are commonly found in viruses, as nonself or foreign. The RNA patterns that mark mRNA as foreign include lack of a 7-methylguanosine cap, absence of 2′O-methylation on the first nucleotide, or double-stranded RNA (dsRNA) structures. As such, proper mRNA capping and conversion of dsRNA to single-stranded RNA (ssRNA) are required for self-mRNA to escape immune detection. The cellular RNA deaminase adenosine deaminase acting on RNA 1 (ADAR1) catalyzes the conversion of adenosine to inosine (A-to-I) in dsRNA structures, resulting in ssRNA that is largely protected from immune sensing [1]. While cellular mRNA is typically not double stranded, dsRNA can arise in cellular mRNA that contains specific repetitive elements. These elements include Alu retroelements, which contain repetitive sequences found largely in introns and the 3′ untranslated regions of mRNA. Alu repeats undergo base pairing, resulting in a dsRNA structure that is a potent activator of PRRs. As such, the A-to-I editing activity of ADAR1 is essential to prevent IFN induction by immunostimulatory dsRNA [2]. Indeed, the autoimmune disease Aicardi–Goutières syndrome (AGS) is associated with mutations in genes encoding dsRNA sensing or processing proteins, such as the dsRNA sensor MDA5 and the cellular RNA deaminase ADAR1 [3].

Recently, the RNA modification N6-methyladenosine (m^6^A) has emerged as another RNA pattern that marks RNA as self. m^6^A can shield mRNA from sensing by RIG-I, a mechanism that has been co-opted by RNA viruses in order to escape immune surveillance [4]. The function of m^6^A on viral RNA is not limited to immune evasion, and it can have both positive and negative impacts on viral infection due to either direct effects on viral RNA or indirect effects on cellular mRNAs that encode factors important for the immune response [4,5]. For example, m^6^A on *IFNB1*, which encodes the cytokine IFN-β, decreases its mRNA half-life, and m^6^A on antiviral mRNAs can influence their expression by increasing translation or decreasing stability. Thus, modifications to RNA, including m^6^A and A-to-I editing, can play important and diverse roles in viral infection and the host innate immune response. Despite this, the full interplay of how these and other RNA modifications orchestrate antiviral signaling remains unclear.

**Fig 1:**
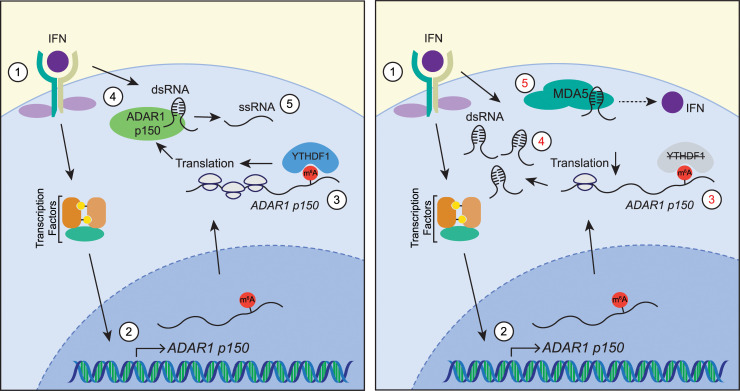
YTHDF1 promotes translation of m^6^A-modified, IFN-induced *ADAR1* to prevent immune activation by self-RNA. (1) IFN signaling, which induces dsRNA, (2) activates the transcription of IFN-stimulated genes, including m^6^A-modified *ADAR1* p150. Left: (3) The m^6^A reader, YTHDF1, promotes the translation of *ADAR1* p150, (4) which then catalyzes A-to-I editing of dsRNA, (5) converting them to ssRNA. Right: (3) In the absence of YTHDF1, *ADAR1* p150 translation is attenuated, (4) leading to reduced editing and the accumulation of dsRNA, (5) which activates the dsRNA sensor MDA5 to induce IFN. ADAR1, adenosine deaminase acting on RNA 1; A-to-I, adenosine-to-inosine; dsRNA, double-stranded RNA; IFN, interferon; MDA5, melanoma differentiation–associated protein 5; m^6^A, N6-methyladenosine; ssRNA, single-stranded RNA; YTHDF1, YTH N6-methyladenosine RNA binding protein 1.

In a new study published in *PLOS Biology*, Terajima and colleagues seek to understand the intersection of m^6^A and A-to-I editing, using glioblastoma cells as a model [6]. Previous studies identified a conserved m^6^A site in the *ADAR1* transcript and an inverse relationship between m^6^A and A-to-I editing, suggesting that a causal link between m^6^A modification on *ADAR1* and A-to-I editing might exist. This new work reveals that the m^6^A RNA–binding protein called YTH N6-methyladenosine RNA binding protein 1 (YTHDF1), which promotes translation of m^6^A-containing mRNAs, binds both isoforms of *ADAR1* to varying degrees, with strong enrichment seen for the IFN-induced p150 isoform and less enrichment for the basally expressed p110 isoform (Fig 1). Contrary to the inverse relationship previously described between m^6^A and A-to-I editing, the expression of the IFN-induced ADAR1 p150 protein was significantly impaired following the depletion of either the m^6^A writer enzymes METTL3 or METTL14 or the m^6^A-binding protein YTHDF1. This indicates that m^6^A deposition and its recognition by YTHDF1 are important for the translation of the *ADAR1* p150 isoform. Consequently, this resulted in a global reduction of A-to-I editing events of cellular mRNA, leading to increased levels of cellular dsRNA and subsequent recognition by the dsRNA sensor MDA5 to induce IFN. These results support the work of others, indicating that MDA5 is activated by self-RNA upon ADAR1 deficiency [2,7,8]. Thus, during the IFN response, the m^6^A-binding protein YTHDF1 enhances ADAR1 p150 expression in glioblastoma cells to facilitate A-to-I editing of self-RNA and prevent activation of dsRNA sensors.

This study by Terajima and colleagues broadens our understanding of how RNA modifications can directly control gene expression and indirectly regulate innate immune signaling, all of which could contribute to autoimmune disorders, leaving the field with much to consider for future study. For example, the depletion of YTHDF1 resulted in increased dsRNA and IFN induction, suggesting that m^6^A and its regulatory proteins could have important roles in preventing autoinflammation. Interestingly, in the cell types tested, this regulation was unique to glioblastoma cells as other cell types did not induce IFN in response to YTHDF1 depletion, suggesting that this might be a mechanism shared in human cells of glial origin. As other studies of YTHDF1 in RNA virus infection have not found that loss of its expression results in broad induction of IFN [4], there are likely important mechanisms that explain these cell type differences. Exploration of the breadth of this phenomenon in different cell types will add to our understanding of how these differences are controlled.

Interestingly, the source of dsRNA produced in response to IFN treatment of glioblastoma cells is also unknown. IFN does not globally alter the frequency of Alu elements, a common source of dsRNA [7,8], although more dsRNA is produced in response to IFN in glioblastoma cells [6]. Thus, it remains to be determined if specific classes of mRNAs with secondary structure are induced by IFN and then edited by ADAR1 p150, as they are in response to bacterial lipopolysaccharide, or if there are RNA processing defects that induce more dsRNA independent of Alu repeats, as seen by others [9,10]. Further, it remains unknown if IFN induces dsRNA in other cell types and how the cell normally resolves this dsRNA to prevent recognition by dsRNA sensors. Ultimately, the work by Terajima and colleagues has provided a platform to define the interplay of RNA modifications and gene expression and how those can impact cellular homeostasis and infection. Further, it adds to the growing body of work revealing the intricate mechanisms of cellular surveillance that distinguish foreign or dysregulated nucleic acids from self-nucleic acids.

## Abbreviations

ADAR1: adenosine deaminase acting on RNA 1
AGS: Aicardi–Goutières syndrome
A-to-I: adenosine-to-inosine
dsRNA: double-stranded RNA
IFN: interferon
MDA5: melanoma differentiation–associated protein 5
m^6^A: N6-methyladenosine
PRR: pattern recognition receptor
RIG: retinoic acid–inducible gene I
ssRNA: single-stranded RNA
YTHDF1: YTH N6-methyladenosine RNA binding protein 1
